# The Effect of Treating Bacterial Vaginosis on Preterm Labor

**DOI:** 10.1080/10647440300025509

**Published:** 2003

**Authors:** Christine C. Tebes, Catherine Lynch, John Sinnott

**Affiliations:** 1 Department of Obstetrics and Gynecology University of South Florida Tampa FL USA; 2 Department of Infectious Diseases University of South Florida PO Box 1289 Tampa FL 33601-1289 USA

## Abstract

*Objective:* Multiple studies suggest that bacterial vaginosis (BV) causes preterm labor; yet its routine treatment remains controversial. In order to help to elucidate this controversy, we performed a thorough review of studies with levels of evidence ranging from I to II–II.

*Methods:*  We searched for all of the studies from the years 1994 to 2001 via Medline’s database, including MD Consult and Ovid Mednet.

*Results:*  Several trials discovered a decrease in the incidence of preterm labor when BV was treated, but most of those trials were performed on women with a history of preterm labor. However, the majority of trials reviewed advise against treatment of a general low-risk obstetric population, as there was no significant decrease in preterm labor.

*Conclusions:* Therefore, based on the above studies and the current guidelines of the Centers for Disease Control and Prevention (CDC), treating pregnant women in high-risk populations who are diagnosed with BV provides the clinician with an opportunity to possibly prevent preterm labor in this population. In nulliparous women without a history of preterm birth, treatment is recommended if other risk factors are present (e.g. gonorrhea or chlamydia). However, in the general low-risk populations, routine screening is not indicated.


*Infect Dis Obstet Gynecol 2003;11:123–129*

### The effect of treating bacterial vaginosis on preterm labor

**Christine C. Tebes[1], Catherine Lynch[1] and John Sinnott[2]**

[1]*Department of Obstetrics and Gynecology, and*
[2]*Department of Infectious Diseases, University of South Florida, Tampa, FL*

***Objective:*** Multiple studies suggest that bacterial vaginosis (BV) causes preterm labor; yet its routine treatment remains controversial. In order to help to elucidate this controversy, we performed a thorough review of studies with levels of evidence ranging from I to II–II.
***Methods:*** We searched for all of the studies from the years 1994 to 2001 via Medline's database, including MD Consult and Ovid Mednet.
***Results:*** Several trials discovered a decrease in the incidence of preterm labor when BV was treated, but most of those trials were performed on women with a history of preterm labor. However, the majority of trials reviewed advise against treatment of a general low-risk obstetric population, as there was no significant decrease in preterm labor.
***Conclusions:*** Therefore, based on the above studies and the current guidelines of the Centers for Disease Control and Prevention (CDC), treating pregnant women in high-risk populations who are diagnosed with BV provides the clinician with an opportunity to possibly prevent preterm labor in this population. In nulliparous women without a history of preterm birth, treatment is recommended if other risk factors are present (e.g. gonorrhea or chlamydia). However, in the general low-risk populations, routine screening is not indicated.

Key words: INFECTION; MOBILUNCUS; ANTIBIOTICS; VAGINITIS; PREMATURITY

Premature births remain a serious problem in the USA, occurring in 11% of all pregnancies. Preterm birth, defined as delivery of an infant before 37 weeks' gestation, is also the leading cause of neonatal mortality and morbidity in the developed world. In the USA alone, an excess of $4 billion[1], or 57% of direct nursery costs[2] can be attributed to the care of premature infants. In addition, infants who survive exhibit an increased risk of long-term morbidity, chronic lung disease, cerebral palsy, developmental delay, and visual and hearing impairment[1,2].

Causes of preterm labor vary, but infection is highly suspect. Up to 80% of early premature births are associated with an intrauterine infection prior to the rupture of membranes. Asymptomatic bacteriuria, *Neisseria gonorrhea*, *Chlamydia trachomatis*, *Trichomonas vaginalis* and bacterial vaginosis (BV) have all been associated with an increased risk of preterm birth[3]. The exception is vaginal yeast infections[4]. Risk factors for preterm delivery are listed in Table 1. Multiple studies suggest that BV is a cause of preterm labor, yet its routine treatment remains controversial[5–8].

In conjunction with preterm delivery, BV in pregnant patients is associated with premature rupture of membranes, infection of the amnion and chorion, histologic chorioamnionitis and infection of amniotic fluid. Flynn *et al.*[5] noted a 60% increase in the risk of preterm delivery in the presence of BV. Another study by Hillier *et al.*[6] associated BV with risk of spontaneous preterm

Correspondence to: John Sinnott, MD, Department of Infectious Diseases, University of South Florida, PO Box 1289, Tampa, FL 33601-1289, USA. Email: jsinnott@tgh.org

© 2003 The Parthenon Publishing Group 

**Table 1** Risk factors for preterm labor[1,2]

| |
|---|
| History of preterm birth |
| Multiple gestation |
| Bacterial vaginosis |
| Urinary tract infection |
| Crack cocaine usage |
| Abruption |
| Second- and third-trimester vaginal bleeding |
| Maternal age under 16 years or over 40 years |
| African–American race |
| History of uterine surgery |
| Smoking |
| Strenuous work |
| Polyhydramnios and oligohydramnios |
| Cervical infections |
| Cervical incompetence |
| Pre-eclampsia |
| Premature preterm rupture of membranes |
| Domestic violence |
| Retained intrauterine device |

birth by a factor of 1.5 to 3 in high-risk women, while other studies suggest that BV almost doubles the risk of spontaneous preterm delivery[7].

Although BV is present in almost 20% of pregnant women, most cases remain asymptomatic, and not all women with the condition will deliver prematurely[8]. It is postulated that there exists a subgroup of high-risk women (e.g. those who have vaginal colonization with *Mycoplasma hominis*). African–American women may also exhibit 200–300% more BV than white populations[7,8]. The exact conditions under which BV directly correlates with preterm labor are unknown[5].

The purpose of this paper is to review the recent literature addressing the association between BV and preterm birth[4].

### METHODS

In order to perform this review, we searched the Medline database, including MD Consult and Ovid Mednet for the years 1994–2001. Some of the search terms included preterm labor, BV, treatment, metronidazole, clindamycin, and vaginitis.

### RESULTS

Hauth *et al.* (Level I study in 1995) treated pregnant women with a history of preterm birth or weight < 50 kg and a positive diagnosis of BV[7]. Treatment consisted of metronidazole (250 mg three times a day for 7 days) and erythromycin (333 mg three times a day for 14 days). The results of this 2:1 double-blind randomization trial revealed a decreased incidence of preterm delivery (< 37 weeks) for the entire study population (odds ratio, OR, 0.48; 95% confidence interval, CI 0.28–0.81), as well as for a subset of patients with a previous preterm delivery (OR, 0.48; 95% CI, 0.25–0.90). Women who were diagnosed with BV at the initial visit (24 weeks) and who received antibiotics rather than placebo presented with fewer preterm deliveries. Since this treatment benefit was only observed at initial examination, the overall results do not support mid-trimester treatment with metronidazole and erythromycin in women at risk for preterm delivery without BV. There are no data to suggest that treatment of low-risk pregnant women with BV decreases the rates of prematurity[7].

Over a period of 3 years, Morales *et al.* (level I study in 1994) screened for BV in women between 13 and 20 weeks with a singleton gestation and a history of preterm birth via a double-blind randomized trial. The 80 women with a positive screen received either metronidazole (250 mg three times a day) or a placebo for 7 days. The study showed a significant decrease in delivery prior to 37 weeks among women taking metronidazole (18%) compared with those on placebo (39%) ($p < 0.05$). In the metronidazole group there were significantly fewer hospital admissions for preterm labor, cases of premature rupture of membranes, and low birth weights[9].

In 1995, McGregor *et al.* (level I study) performed a prospective, controlled treatment trial of 1260 subjects to study the effect of clindamycin on pregnant women with BV. Women who were treated with 300 mg of clindamycin orally twice daily for 7 days showed a reduction in preterm birth (relative risk 0.5; 95% CI, 0.3–0.9), and the authors recommended that women at risk for preterm birth with BV should be screened and treated[1].

In 1997, McDonald *et al.*[10] conducted a multicenter, randomized, placebo-controlled trial of 879 women at 19 weeks' gestation, but failed to demonstrate a reduced preterm birth rate among pregnant women with BV or those with a heavy growth of *Gardnerella vaginalis* (level I). The intention-to-treat analysis showed no difference between the treatment and placebo groups in overall preterm birth (31/429 [7.2%] vs. 32/428 [7.5%]) or spontaneous preterm birth rate (20/429 [4.7%] vs. 24/428 [5.6%]). However, among women with a previous preterm birth, those treated with oral metronidazole (400 mg twice daily for 2 days at 24 weeks' gestation, and again at 29 weeks) demonstrated a marked reduction in spontaneous preterm birth rate (OR, 0.14; 95% CI, 0.01–0.84)[10].

A meta-analysis from the Cochrane database determined that preterm birth rates did not differ significantly between treated and non-treated pregnant patients with BV (OR, 0.78; 95% CI, 0.60–1.02), yet a subgroup of women with a previous preterm birth demonstrated a significant decrease in the incidence of preterm birth, with an odds ratio of 0.37 (95% CI, 0.23–0.60)[3]. This meta-analysis of 1504 women from a total of five trials using amoxicillin, clindamycin and metronidazole did not recommend screening and treating all pregnant women for BV in order to prevent preterm birth.

Carey *et al.*[11] recently conducted a randomized, double-blind clinical trial of the use of metronidazole to treat asymptomatic BV. Treatment did not reduce the frequency of delivery before 37 weeks' gestation (relative risk in the metronidazole group, 1.0; 95% CI, 0.8–1.2). This clinical trial differed from previous attempts because it was larger (1953 subjects) and it studied the general obstetrical population, not just women with a history of preterm delivery. Treatment involved a shorter course of metronidazole therapy with two 2 g doses during a 48-hour period, and a second treatment of the women between 24 and 30 weeks' gestation. These researchers recommended the longer course of therapy to eradicate upper genital tract organisms[11].

The results of this study supported those of McDonald *et al.*[10] in that metronidazole did not reduce preterm delivery among the general population of pregnant women, nor did it reduce hospital admissions for preterm labor or preterm premature rupture of the membranes, postpartum endometritis, clinical intra-amniotic infections, vaginal infections serious enough to require treatment, and treatment with tocolytic drugs. The placebo and metronidazole groups did not differ with regard to incidence of admissions to the neonatal intensive-care unit, passage of meconium, or fetal death/neonatal death. This study found that routine screening of pregnant women for asymptomatic BV and treating the latter with a short course of orally administered metronidazole did not reduce the risk of preterm birth, despite its effectiveness in eradicating BV. Studies do not support the use of metronidazole treatment in pregnant women with asymptomatic BV who are considered to be at either high or low risk for preterm delivery[11]. Studies by McDonald *et al.*[10], Morales *et al.*[9] and Hauth *et al.*[7] did find evidence of a decreased incidence of recurrent preterm delivery among women with a prior preterm delivery treated with metronidazole or metronidazole/erythromycin.

The findings of Joesoef *et al.*[12] concur with those of Carey *et al.*[11]. This multicenter, double-blind, randomized, placebo-controlled trial failed to demonstrate a reduction in preterm delivery (level I, 1995) in 745 women between 14 and 26 weeks' gestation who were diagnosed with BV (via Gram stain score > 6) with 2% clindamycin vaginal cream or with a placebo cream for 7 days. Although 15% of the women treated with clindamycin had a preterm delivery, only 13.5% of placebo patients did (OR, 1.1; 95% CI, 0.7–1.7). The authors proposed that the increased frequency of preterm delivery might be caused by a transient increase in vaginal colonization by *Escherichi coli* and *Enterococcus* 1 month after therapy. Since *E. coli* is linked to an increased risk of preterm delivery, this may explain the value of 15%, compared with the 13.5% difference found in the study by Joesoef *et al.*[12].

In 1994, McGregor *et al.*[13] evaluated 271 women between 16 and 27 weeks' gestation in a double-blind trial (level I). Women who were diagnosed with BV were treated with 2% clindamycin vaginal cream or placebo for 7 days. Although 2% clindamycin vaginal cream was effective in treating BV during pregnancy ($p = 0.001$) it was not permanent, as the condition gradually returned, and treatment did not reduce the risk of prematurity during the second trimester.

Possible reasons cited include the inadequate power of the study, the timing of treatment, and the use of local vaginal treatment[13].

In 2001, Kekki *et al.*[14] performed a multicenter, randomized, double-blind, placebo-controlled trial (level I) which showed that the treatment of BV in early pregnancy with vaginal clindamycin for 7 days did not decrease the rate of preterm deliveries or peripartum infections. In 375 randomized subjects, preterm delivery occurred in 5% of the clindamycin group and 4% of the placebo group (OR, 1.3; 95% CI, 0.5–3.5). The efficacy of intravaginal clindamycin can be as high as 90% in studies on non-pregnant women, and is similar to that of oral metronidazole. The subjects included a low-risk population of healthy women with singleton pregnancies and without a history of preterm delivery[14]. The results are similar to the conclusion of McGregor *et al.*[13] that topical treatment in early pregnancy reduced vaginal fluid mucinase and sialidase, but failed to reduce the rate of preterm births[14].

A recent randomized controlled trial by Kurkinen-Raty *et al.*[15] assessed the efficacy of vaginal clindamycin in reducing preterm birth (level I). Of the 1956 women without a history of preterm delivery who were screened at the first antenatal visit (gestational week 12), 143 women tested positive for BV. After randomization and treatment with clindamycin or placebo, the preterm birth rate in the clindamycin group was 13.7%, compared with 6.0% in the placebo group (OR, 2.5; 95% CI, 0.6–10). This study therefore supports the evidence that vaginal clindamycin treatment of BV in the first trimester does not reduce the risk of preterm birth[15].

In 1999, French *et al.*[16] evaluated the association between BV, first-trimester vaginal bleeding and preterm labor in 1100 pregnant women who were enrolled in a prospective observational study (level II–II). It was determined that treatment of BV with clindamycin (300 mg orally twice daily for 7 days) significantly reduced the risks of preterm birth among women without first-trimester bleeding (relative risk, 0.37; 95% CI, 0.16–0.88). Although the overall population of women with both BV and first-trimester vaginal bleeding experienced reductions in preterm birth, the finding was not statistically significant (relative risk, 0.52; 95% CI, 0.18–1.55)[16].

Several authors have proposed that the timing of the diagnosis and/or treatment is important if preterm labor is to be decreased. A level II–II study by Meis *et al.*[17] highlighted the effect of timing of diagnosis on BV and the incidence of preterm labor. Women who tested negative at 24 weeks but were positive when tested again at 28 weeks had the highest likelihood of preterm birth (OR, 2.53; 95% CI, 1.32–4.85; $p = 0.005$)[17].

Riduan *et al.*[18] found the opposite to be true (level II–I trial). Women who tested positive for BV at 24 weeks and received antibiotics showed a significantly decreased incidence of preterm birth (OR, 0.44; 95% CI, 0.11–1.91). The authors found that the presence of BV at 16 weeks was more predictive of preterm delivery than its presence at 28 to 32 weeks[18].

Table 2 provides a concise summary of the articles reviewed above.

### DISCUSSION

Preterm labor may be classified as either physiologic or pathologic. Physiologic preterm labor describes a normal initiating factor that occurs too early in pregnancy, while pathologic preterm labor results from an abnormal initiating factor with timing being a distinguishing factor. The earlier in pregnancy preterm labor occurs, the more likely it is that a pathologic etiology exists. Prior to 16 weeks' gestation, BV is related to preterm delivery by a risk factor of 5 to 7.5. After 26 weeks' gestation, the risk factor drops to 1.4 to 1.9[19,20].

BV, an overgrowth of anaerobic species that produce protease, phospholipase A2 and collagenases in the vagina[21], is found in 800 000 pregnant women each year[11]. There is disruption of the vaginal ecosystem that results in increased levels of anaerobes. BV alters the vaginal flora by decreasing the number of hydrogen-peroxide-producing *Lactobacillus acidophilus* organisms. Consequently, the levels of *G. vaginalis*, *M. hominis*, and *Mobiluncus* species increase rather than remaining in their normal state of suppression. The metabolic by-products of these

**Table 2** Studies that have evaluated the effect of treatment of bacterial vaginosis on the incidence of preterm labor

| *Author* | *Year* | *Study type* | *Effect on preterm labor* | *Drug* | *Level of evidence* |
|---|---|---|---|---|---|
| Hauth *et al.*[7] | 1995 | Double-blind | Decreased in high-risk | Metronidazole/ erythromycin | Level I |
| Morales *et al.*[9] | 1994 | Placebo controlled | Decreased in high-risk | Metronidazole | Level I |
| McGregor *et al.*[1] | 1995 | Prospective controlled treatment trial | Decreased in women at high risk | Clindamycin | Level I |
| McDonald *et al.*[10] | 1997 | Randomized, placebo, controlled | Decreased only in subset with a history of preterm labor | Metronidazole | Level I |
| Cochrane database[3] | 1991 to 2001 | Meta-analysis of controlled trials | No decrease in general population, but a decrease in those at high risk | Amoxicillin, metronidazole and/or clindamycin | Level I |
| Joesoef *et al.*[12] | 1995 | Double-blind, randomized, placebo | No decrease in low-risk population | Metronidazole | Level I |
| Carey *et al.*[11] | 2000 | Randomized, double-blind | Asymptomatic general population without decrease | Metronidazole (short course) | Level I |
| McGregor *et al.*[13] | 1994 | Randomized, double-blind | No decrease in women at 16 to 27 weeks' gestation | Clindamycin | Level I |
| Kekki *et al.*[14] | 2001 | Double-blind, placebo | No decrease in low-risk population | Clindamycin | Level I |
| Kurkinen-Raty *et al.*[15] | 2000 | Randomized, controlled | No significant decrease in low-risk population | Clindamycin | Level I |
| French *et al.*[16] | 1999 | Prospective, observational | Decreased in low-risk population, but not statistically significant | Clindamycin | Level II–II |

organisms include amines, which increase the vaginal pH, and exfoliation of vaginal epithelial cells results[22].

Although the exact mechanism is not known, studies have shown that BV can cause infection of the upper genital tract, which acts as a premature birth trigger[6]. BV and *T. vaginalis* are also associated with many microorganisms that produce phospholipase A2 and C or phospholipase-like activity, and affected patients show increased levels of sialidase, phospholipase A2, prostaglandin E2 and interleukin–1(beta). The rise in levels of these enzymes may result in the decidual or fetal membrane cell fatty acid tissue stores releasing arachidonic acid[2], a precursor of the uterotonic prostaglandins.

Other explanations of an association between BV and preterm labor include activation of fetal and/or maternal inflammatory responses or proteolytic enzymes. Elevated vaginal or cervical levels of endotoxin, mucinase, sialidase and interleukin-1(alpha) are found in women with BV, which suggests that the microorganisms produce cytokines[6]. These cytokines and the release of interleukin-1(beta) and tumor necrosis factor induce cyclooxygenase II, an enzyme that produces prostaglandins involved in parturition. Proteolytic enzymes that may overcome maternal mucous membrane defenses and impair fetal membrane strength and elasticity include collagenases, immunoglobulin A proteases, elastases, mucinases and/or sialidases[2].

Several studies have concluded that screening and treating for BV is futile, and one of the largest clinical trials to date found no difference in the treated low-risk pregnant population compared with the placebo[11]. However, since the clinical diagnosis of BV need not be symptomatic, screening all pregnant women who have risk factors may be unnecessary and not cost-effective[21], while the most advantageous time to screen and the optimum dosage of antibiotic is uncertain.

Although the *The Cochrane Library's* review is an excellent resource, our paper differs in that we have investigated different aspects of this approach. We have included not only level I but also level II studies. Furthermore, we included two studies that investigated the effects of timing on the treatment of BV[17,18]. However, ultimately our conclusion is similar to that of the Cochrane review[3].

To the clinicians who decide to screen for and treat BV, the most effective antibiotic appears to be oral metronidazole, which can be safely administered during pregnancy. Vaginal clindamycin therapy has shown slight but statistically non-significant increases in preterm birth rate for the reasons discussed previously[23].

Analysis of the reviewed studies supports the current guidelines of the Center for Disease Control. Treating pregnant women in high-risk populations who have been diagnosed with BV can prevent preterm labor in these individuals. In nulliparous women without a history of preterm birth, the recommendation is to treat if other risk factors, such as gonorrhea or chlamydia, are present.

### REFERENCES

1. McGregor JA, French JI, Parker R, *et al*. Prevention of premature birth by screening and treatment for common genital tract infections: results of a prospective controlled evaluation. *Am J Obstet Gynecol* 1995;173:157–67
2. Andolsek KM, Kelton GM. Risk assessment. *Prim Care* 2000;27:71–103
3. Brocklehurst P, Hannah M, McDonald H. Interventions for treating bacterial vaginosis in pregnancy. *The Cochrane Library. Issue 4*. Oxford: Update Software, 2001
4. Goldenberg RL, Rouse DJ. Prevention of premature birth. *N Engl J Med* 2001;339:313–20
5. Flynn CA, Helwig AL, Meurer LN. Bacterial vaginosis in pregnancy and the risk of prematurity: a meta-analysis. *J Fam Pract* 1999;48:885–92
6. Hillier SI, Nugent RP, Eschenbach DA, *et al*. Association between bacterial vaginosis and preterm delivery of a low-birth-weight infant. *N Engl J Med* 1995;333:1737–42
7. Hauth JC, Goldenberg RL, Andrews WW, *et al*. Reduced incidence of preterm delivery with metronidazole and erythromycin in women with bacterial vaginosis. *N Engl J Med* 1995;333:1732–6
8. Lamont RF. Antibiotics for the prevention of preterm birth. *N Engl J Med* 2000;342:581–3
9. Morales WJ, Schorr S, Albritton J. Effect of metronidazole in patients with preterm birth in preceding pregnancy and bacterial vaginosis: a placebo-controlled, double-blind study. *Am J Obstet Gynecol* 1994;171:345–9
10. McDonald HM, O'Loughlin JA, Vigneswaran R, *et al*. Impact of metronidazole therapy on preterm birth in women with bacterial vaginosis flora (*Gardnerella vaginalis*): a randomized, placebo controlled trial. *Br J Obstet Gynaecol* 1997;104:1391–7
11. Carey JC, Klebanoff MA, Hauth JC, *et al*. Metronidazole to prevent preterm delivery in pregnant women with asymptomatic bacterial vaginosis. *N Engl J Med* 2000;342:534–40
12. Joesoef MR, Hillier SL, Wiknjosastro G, *et al*. Intravaginal clindamycin treatment for bacterial vaginosis: effects on preterm delivery and low birth weight. *Am J Obstet Gynecol* 1995;173:1527–31
13. McGregor JA, French JI, Jones W, *et al*. Bacterial vaginosis is associated with prematurity and vaginal fluid mucinase and sialidase: results of a controlled trial of topical clindamycin cream. *Am J Obstet Gynecol* 1994:170:1048–59
14. Kekki M, Kurki T, Pelkonen J, *et al*. Vaginal clindamycin in preventing preterm birth and peripartal infections in asymptomatic women with bacterial vaginosis: a randomized, controlled trial. *Obstet Gynecol* 2001;97:643–8
15. Kurkinen-Raty M, Vuopala S, Koskela M, *et al*. A randomized, controlled trial of vaginal clindamycin for early pregnancy bacterial vaginosis. *Br J Obstet Gynaecol* 2000;107:1427–32
16. French JI, McGregor JA, Draper D, *et al*. Gestational bleeding, bacterial vaginosis and common reproductive tract infections: risk for preterm birth and benefit of treatment. *Obstet Gynecol* 1999;93:715–24
17. Meis PJ, Goldenberg RL, Mercer B. The preterm prediction study: significance of vaginal infections. *Am J Obstet Gynecol* 1995;173:1231–5
18. Riduan JM, Hillier SL, Utomo B, *et al*. Bacterial vaginosis and prematurity in Indonesia: association in early and late pregnancy. *Am J Obstet Gynecol* 1993;169:175–8

19. Kurki T, Sivonen A, Renkonen OV, *et al.* Bacterial vaginosis in early pregnancy and pregnancy outcome. *Obstet Gynecol* 1992;80:173–7
20. Hay PE, Lamont RF, Taylor-Robinson D, *et al.* Abnormal bacterial colonization of the genital tract and subsequent preterm delivery and late miscarriage. *Br Med J* 1994;308:295–8
21. McCoy M, Katz C, Vern L, *et al.* Bacterial vaginosis in pregnancy: an approach for the 1990s. *Obstet Gynecol Surv* 1995;50:482–8
22. Egan ME, Lipsky MS. Diagnosis of vaginitis. *Am Fam Physician* 2000;62:1095–104
23. Burtin P, Taddio A, Ariburnu O, *et al.* Safety of metronidazole in pregnancy: a meta-analysis. *Am J Obstet Gynecol* 1995;172:525–9

Received 11/06/02; Accepted 05/02/03
